# Patient-Reported Nonadherence with Glaucoma Therapy

**DOI:** 10.1089/jop.2018.0134

**Published:** 2019-05-07

**Authors:** Christian Wolfram, Erik Stahlberg, Norbert Pfeiffer

**Affiliations:** ^1^ Department of Ophthalmology, University Medical Center Mainz, Mainz, Germany.; ^2^ Department of Ophthalmology, University Medical Center Hamburg-Eppendorf, Mainz, Germany.; ^3^ Department for Radiology and Nuclear Medicine, University Hospital of Schleswig Holstein, Campus Luebeck, Mainz, Germany.

**Keywords:** glaucoma, nonadherence, adherence, preservatives, outcomes, side effects

## Abstract

***Purpose:*** Effective glaucoma therapy relies to a great extent on the patients' ability to regularly self-administer eye drops. This study aimed to assess self-reported nonadherence and to identify potential barriers to adherence in glaucoma patients.

***Methods:*** Participants completed a 16-item questionnaire, designed to examine nonadherence rate and assess the therapy experience. Inclusion criteria stipulated treatment duration of at least 1 year. Nonadherence was defined as missing ≥5% of the prescribed pressure-lowering eye drops doses.

***Results:*** In total, 201 glaucoma patients aged 24–88 years were included. Mean treatment duration was 9.4 years. Nonadherence was reported by 30.3% of participants and 69.7% were reported to be adherent. Individuals who experienced side effects reported higher levels of nonadherence than those who did not (37.6% vs. 18.4%; *P* = 0.004). Eye drops with preservatives were used by 84.1% of participants, 11.9% were on combined preservative and preservative-free treatment, and 4.0% on preservative-free medication only. Self-reported nonadherence levels were 32.0%, 25.0%, and 12.5%, respectively, for each of these groups. Men reported higher rates of nonadherence than women (36.8% vs. 24.5%; *P* = 0.066). Age, social status, history of migration, severity of disease, and fear of blindness were not associated with significant differences in nonadherence levels.

***Conclusions:*** Nonadherence with glaucoma therapy is a significant barrier to therapeutic success for approximately one-third of patients. Nonadherence may be reduced if side effects are avoided. Preservative-free products may provide adherence benefits. The patient experience should be a key consideration when selecting appropriate treatments, to reduce nonadherence and optimize outcomes.

## Introduction

Effective and regular self-administration of eye drops is crucial for therapy success in glaucoma.^1^ For any chronic disease, including glaucoma, patients may experience difficulty in adhering constantly to a long-term treatment. Research in different chronic pathological settings, such as in inflammatory bowel disease, has demonstrated that therapy adherence may be limited.^2^ A review of adherence research over the past 3 decades concluded that between 30% and 50% of all patients complied poorly with their therapy, irrespective of disease, prognosis, and setting.^3^

The term “adherence” has recently replaced the formerly used term “compliance” in the literature, as this had been criticized for placing the patient in too passive a position regarding responsibility for their ongoing therapy.^4^ Adherence is generally considered to be the degree to which treatment goals and pathways are met, as mutually defined by the patient and their physician, through ongoing and consistent administration of clinical interventions (pharmacological and nonpharmacological).

For glaucoma, a meta-analysis of adherence studies demonstrated a wide range of adherence levels, varying between 5% and 80%, which was also due to the fact that no common definition of adherence was in place.^5^ However, even with respect to inconsistent monitoring strategies and adherence measures, most studies concerning nonadherence in glaucoma met the abovementioned range or were slightly lower.^6^

Perfect adherence for all patients is impossible to achieve. It is therefore important to study the drivers and barriers in the daily routine for patients and to identify critical settings or high-risk patients who tend to demonstrate less adherent behavior. Previous studies described younger patients and men, in addition to patients at earlier stages of the disease, to be less adherent.^7–9^ Furthermore, a lower general health status, and particularly psychiatric conditions such as depression were found to reduce adherence, whereas the frequency of dosing and the duration of treatment appeared to have no major influence.^3,8,10^

Levels of nonadherence may also depend on the medication itself. It has been demonstrated that preservatives in the eye drops can increase the patients' discomfort with the therapy and may lead to ocular surface problems.^11^ In contrast, preservative-free medications were associated with fewer adverse effects concerning the corneal epithelium, which may reduce the overall prevalence of side effects.^12–14^ In a Dutch study, 94% of glaucoma patients were reported to use preserved medications; 44% of the patients experienced side effects and 38% used artificial tears regularly.^15^ The influence of preservative-containing and preservative-free medications on nonadherence rates should therefore be investigated.

The main goal of this study was to gain an understanding of current nonadherence levels concerning self-administered glaucoma therapies, using glaucoma patients attending a German university eye clinic as a sample population, and to investigate potential factors that may improve or otherwise impact nonadherence.

## Methods

### Study population

Study participants were recruited from the Department of Ophthalmology at the University Medical Center Mainz in southwest Germany in 2014. Glaucoma patients with a history of at least 1 year of treatment with intraocular pressure-lowering medications and sufficient knowledge of the German language were included. All study subjects provided informed consent. The study was approved by the Ethical Committee of the Regional Association of Physicians (*Landesärztekammer*) in Rhineland-Palatine in January 2014 and complied with the ethical principles of the Declaration of Helsinki for all human or animal experimental investigations.

### Study design

Self-reported nonadherence with glaucoma therapies was assessed via a bespoke tailored patient questionnaire. Glaucoma patients were asked to complete a questionnaire of 16 items about current and previous medications, the occurrence and kind of side effects they had encountered, frequency of medication use, and the frequency and causes of nonadherence. According to the definition of self-reported adherence used in the European Glaucoma Prevention Study (EGPS), adherence was defined as taking at least 95% of the prescribed applications and nonadherence as missing 5% or more.^16,17^

Additional information was sought concerning the severity and duration of the disease, need for assistance in administering eye drops, visual ability to drive a car, and fear of becoming blind.

Sociodemographic data regarding age, gender, education, history of migration, and urban or rural living situation were used to analyze the sample descriptively and enabled subgroup analyses. For the differentiation of 3 different social levels (upper, middle, and lower), a status index was calculated from the information provided by participants concerning educational and professional status.^18^

A history of migration was defined as having at least 1 parent of nondomestic decent. The threshold of urban background was defined as living in a city of 50,000 or more inhabitants.

### Statistical analysis

To calculate the frequency distribution and to provide a picture of the interrelation of variable groups (e.g., bivariate correlation for preservative vs. nonpreservative group) crosstabs were used. Furthermore, the differences between the groups were tested by using the Pearson's chi-squared or Fisher's exact test, respectively. To measure the relationship between adherence and several subgroup variables such as preservative or nonpreservative medication multivariable logistic regression was performed. In this context the *P* values, odds-ratio, and 95% confidence intervals were calculated. Statistical significance was set at *P* < 0.05. Statistical evaluation of the data was performed by using dedicated statistical software (SPSS™ 22 for Windows; IBM, Armonk, NY).

## Results

### Participant demographics

In total, 201 study participants were recruited and completed the study questionnaire. The mean age of the participants was 65.6 years (range 24–88 years). There was an equal distribution of female and male participants (Table 1). Approximately two-thirds were living in a rural environment and 17.4% had a background of migration in the family (Table 1). Distribution across the 3 defined social levels (upper, middle, and lower social status) was roughly equal (Table 1).

**Table 1.** Sociodemographic Data Concerning the Study Population

	*Participants,* n *(%)*
Total number of participants	201
Women	106 (52.7)
Men	95 (47.3)
Urban background^a^	64 (31.8)
Rural background	137 (68.2)
Background of migration^b^	35 (17.4)
No background of migration	166 (82.6)
Upper social status^c^	56 (27.9)
Middle social status	76 (37.8)
Lower social status	69 (34.3)

^a^ Urban background defined as coming from a city of at least 50.000 inhabitants.

^b^ Migration background defined as at least 1 parent coming from a foreign country.

^c^ Social status calculated by an index out of educational and professional qualification and status.

### Participant characteristics: disease and treatment-related factors

On average, participants had been diagnosed with glaucoma for 9.9 years (range 1–50 years) and mean duration of treatment, to date, was 9.4 years (Table 2). Almost all patients (98.0%) reported that they were able to instill their eye drops without assistance.

**Table 2.** Participant Characteristics: Disease/Treatment-Related Factors

	*Value,* n *(%)*
Mean disease duration (years)	9.9
Mean treatment duration (years)	9.4
No assistance for taking eye drops	197 (98.0)
Need for assistance	4 (2.0)
Medication with preservatives	169 (84.1)
Combined medication with and without preservatives	24 (11.9)
Preservative-free medication	8 (4.0)
Experience of side effects	125 (62.2)
No experience of side effects	76 (37.8)
Reported therapy switch	73 (36.3)
No therapy switch	128 (63.7)
Unable to drive a vehicle due to visual disability	30 (14.9)
Strong or very strong fear of blindness	106 (52.8)

Overall, 84.1% of patients were on treatment that contained preservatives, 11.9% received a combination regimen that included both treatments containing preservative and preservative-free products, and 4.0% were on preservative-free medication only (Table 2). In total, 36.3% of study participants reported to have changed their therapy in the past.

The majority of patients had sufficient visual function to drive a vehicle, but 14.9% were unable to drive due to a visual disability (Table 2). Four participants (1.9%) had no driver's license. More than half of the study participants reported having a strong or very strong fear of blindness (52.8%) (Table 2). Only 8% denied fearing blindness.

**FIG. 1.**
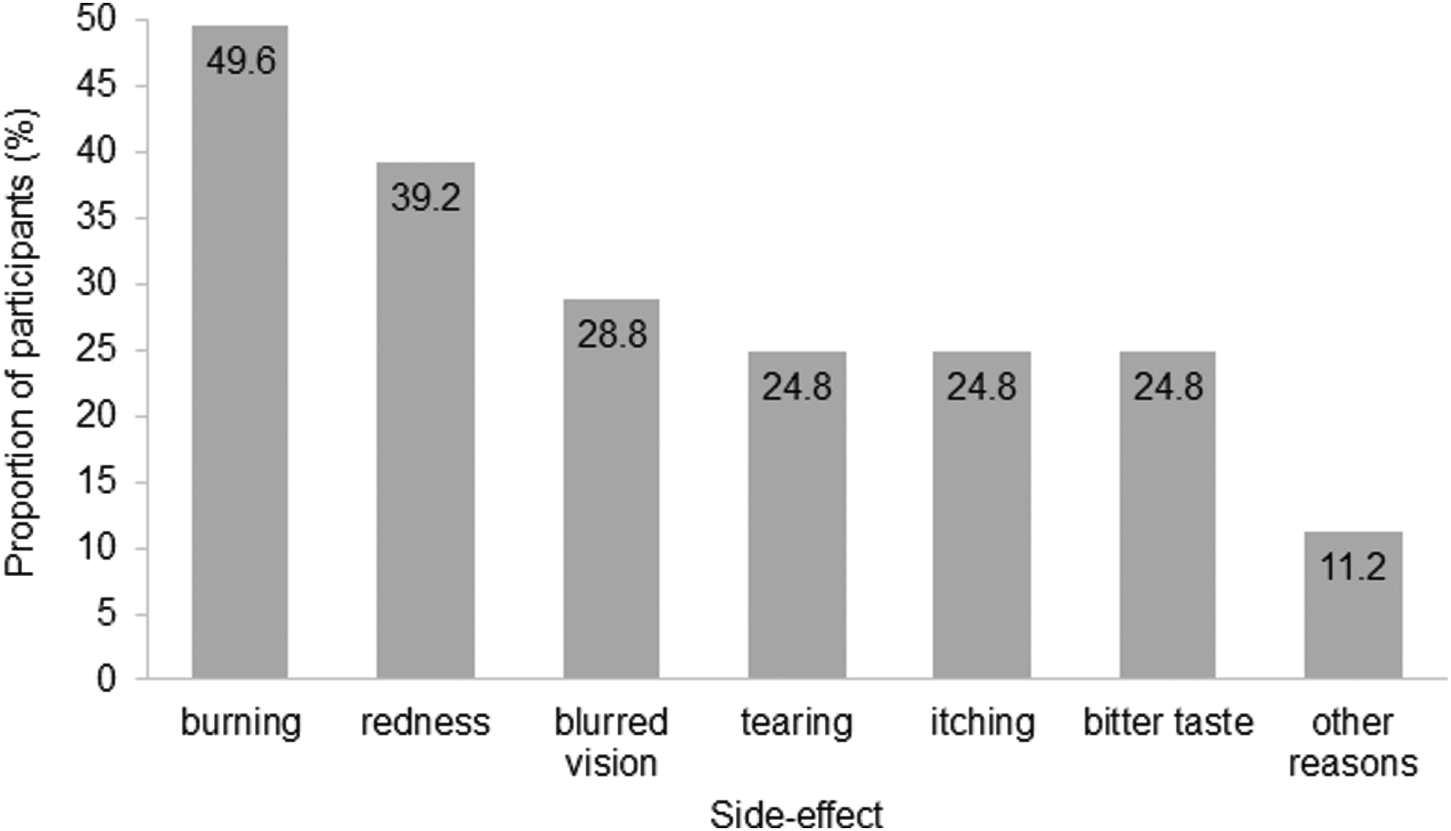
Proportion of participants (%) reporting treatment-related side effects.

Most patients (62.2%) had experienced side effects associated with their topical glaucoma medication (Table 2). Of these, burning was the most commonly reported (49.6%), followed by redness (39.2%), blurred vision (28.8%), tearing, itching (24.8%), and a bitter taste (24.8%) (Fig. 1). Other side effects comprised dizziness, headaches, and dryness of the mouth (11.2%) (Fig. 1).

### Self-reported nonadherence and disease/treatment-related factors

Overall, self-reported nonadherence with the prescribed treatment regimen was reported by 30.3% of the participants and 69.7% were adherent, according to the definition used (Table 3). The ability to drive a car did not influence the nonadherence rate.

**Table 3.** Nonadherence Rates with Respect to Disease/Treatment-Related Factors

	*Nonadherence (%)*	P
Overall nonadherence	30.3	
Experience of side effects	37.6	0.004
No side effects	18.4	
Medication with preservatives	32.0	0.036
Combined medication (with and without preservatives)	25.0	
Preservative-free medication	12.5	
Reported therapy switch	15.1	0.014
No therapy switch	39.1	
No fear of blindness	18.7	0.560
Low fear of blindness	33.3	
Moderate fear of blindness	25.0	
Strong fear of blindness	36.7	
Very strong fear of blindness	28.3	

#### Treatment-related side effects and nonadherence

Self-reported nonadherence was significantly higher in individuals who had experienced at least 1 side effect associated with their glaucoma medication, compared with those who had not encountered treatment-related side effects (37.6% vs. 18.4%; *P* = 0.004) (Table 3).

#### Use of preservatives in medication

The use of preservatives in glaucoma medication was associated with a higher nonadherence rate within the study population (Table 3). Nonadherence rates reached 32.0% among participants taking medication containing preservatives, while just 12.5% nonadherence was observed in those using preservative-free treatments (Table 3). The use of regimens containing a combination of preservative-free and preservative-containing treatments was associated with a 25.0% nonadherence rate (Table 3).

#### Treatment switch

Of the 201 participants included in the study, 73 reported a treatment switch to an alternative treatment since diagnosis. In total, 16 individuals had been moved over to a preservative-free medication and 57 were changed to a preservative-containing regimen. Overall, a switch of medication was associated with a significant reduction in nonadherence concerning current therapy; participants who had not switched demonstrated 39.1% nonadherence and those who changed medications reported 15.1% nonadherence (*P* = 0.014) (Table 3).

Although numbers were relatively small, the majority of participants who had been nonadherent with previous therapies reported improvements in their ability to adhere to their new treatment regimen when switched to either a preservative-free (25.5% nonadherence) or preservative containing (35.7% nonadherence) therapy.

#### Fear of blindness

Those reporting a fear of blindness (at all levels) were less likely to be adherent with medication, compared with participants with no fear of blindness, although no statistically significant differences were observed concerning the level of anxiety associated with potential blindness and nonadherence (Table 3).

### Subgroup analysis

Men demonstrated a higher rate of self-reported nonadherence (36.8%), compared with women (24.5%), but this difference was not statistically significant (*P* = 0.066) (Table 4).

**Table 4.** Nonadherence Rates and Participant Subgroups

	*Nonadherence (%)*	P
Overall nonadherence	30.3	
Men	36.8	0.066
Women	24.5	
History of migration	31.4	0.843
No migration	30.1	
Urban living environment	35.9	0.233
Rural living environment	27.7	
Upper social status	28.6	0.609
Middle social status	27.6	
Lower social status	34.8	

Age did not appear to be significantly related to therapy adherence or nonadherence. The mean age of nonadherent participants was 64.11 years, with a standard deviation (SD) of 11.23, and the mean age of adherent individuals was 66.26 years (SD = 13.01) (*P* = 0.83).

Background of migration did not influence nonadherence levels (Table 4). Patients from an urban background were slightly less adherent (35.9% nonadherence) than those from a rural living environment (27.7% nonadherence). Lower social status tended to be associated with higher levels of nonadherence (34.8%), compared with the middle (27.6%) or upper (28.6%) social levels. However, these differences were not statistically significant (*P* = 0.609).

Among those who had missed their therapy, 40.8% said that they had forgotten their medication, 13.9% cited side effects, and only 2.0% highlighted multiple medications as reasons for nonadherence.

## Discussion

### Self-reported nonadherence rates in glaucoma patients

In this study investigating nonadherence in glaucoma patients, >30% of patients reported missing instillation of 5% or more of their regular treatment. This nonadherence rate is consistent with the expected range, reported in similar studies concerning self-reported adherence and nonadherence, even where definitions of adherence vary.^3,17,19–22^

Nonadherence data may be captured through a number of means; self-reporting, objective measurement using electronic monitoring systems and health insurance claims. The self-reported findings presented in this study are in line with previous glaucoma studies, despite variations concerning the tools and methodologies used.^6,23,24^ Studies using health insurance claims data showed that nonadherence rates were comparable with self-reported results. In general, our findings and most results from the literature suggest that clinicians should expect overall nonadherence rate within a range of 25% −40% for self-administered glaucoma treatments.^25^

### Treatment-related factors affecting nonadherence

The most significant factor associated with nonadherence in this study was the presence of side effects. Burning, redness, and blurred vision were the most commonly reported side effects. The influence of side effects concerning treatment nonadherence has not been established or fully investigated in previous studies.^3,8,14,26^ In the past, side effects were either poorly reported by patients as a reason for nonadherence or simply tolerated, at least at a lower level of disturbance.^3,8,27^ In contrast, the vast majority of glaucoma patients reported a high degree of satisfaction with their therapy, even when they did experience side effects.^15^ In this study, 37.6% of the participants who experienced side effects were not adherent with their glaucoma therapy regimen. In the absence of side effects, nonadherence was significantly reduced to 18.4% (*P* = 0.004), indicating that patients are likely to benefit from the avoidance of even minor adverse events.

Our results highlight a number of potential drivers that may reduce nonadherence and enhance adherence, including choice of therapy. Individuals receiving preservative-free medication demonstrated lower rates of nonadherence (12.5%), compared with those taking preservative-containing medication (32.0%) or a combined regimen of preservative-free and preservative-containing treatment (25.0%). It has previously been shown that monotherapies, particularly with prostaglandins, and simplified treatment regimens lead to better adherence and persistence.^14,25^ Preservative-free products lower corneal toxicity, reduce loss of epithelial cells, and lower the risk of ocular surface diseases, which may explain the better adherence among our study participants.^12,13^ The proportion of patients prescribed a combination of both preserved and preservative-free treatments was surprisingly high, indicating that the influence of each type of treatment on aspects of nonadherence may not have been a factor that physicians considered important when selecting medication or perhaps they may not have been aware of it.

This study also demonstrated that a change in medication tended to be associated with improvements in nonadherence levels, and indicated that moving to preservative-free therapies may be of particular benefit regarding adherence with self-administered treatments.

### Sociodemographic factors

Minor differences were observed regarding reporting of nonadherence when examining age, gender, social status, and local or migration background. However, these were not statistically significant. This is consistent with other studies that also described sociodemographic factors and gender to be less important influencers of nonadherence than medical and behavioral factors.^3,9,10^

### Implications for clinical practice

These results indicate that the treating physician is likely to have a crucial role in influencing patient adherence and nonadherence with self-administered glaucoma therapy.^28,29^ Previous studies revealed that physicians generally estimated higher adherences rates than patients actually achieved.^21,23^ It was also shown that patients often did not tell their physicians about side effects unless these were intolerable.^27^ Up to 33% of patients may have difficulties in properly instilling eye drops.^22,30^ It is, therefore, necessary to sharpen the focus among ophthalmologists on the potential challenges that patients may experience with their treatment and to actively inquire about such problems.

Physicians may also enhance adherence through support and education. Although research to date, including a Cochrane review, has been unable to identify and recommend a specific intervention that is guaranteed to improve adherence with self-administered ocular therapies, it has been found repeatedly that a key element for adherence is the patients' self-efficacy and the need to strengthen intrinsic factors for the individual patient.^31–34^ Surprisingly, it is not necessarily knowledge about the disease and treatment that may improve adherence, but rather the individual understanding and conviction that regular treatment is necessary and helpful to preserve vision.^35^ Successful treatment of glaucoma should not focus on general information alone, but on the individual's motivation for the therapy.

The most common reason for nonadherence in this study was that individuals had simply forgotten to take their medicine (40.8% of nonadherent participants), which has been found previously to be a major obstacle for the treatment of glaucoma.^19,34,36^ Adherence levels may be improved through application of tools, such as reminder systems and dosing aids, which are probably most effective in combination with educational and behavioral approaches.^31,34,36–38^

### Study limitations and opportunities for future research

A key limitation in adherence research is the lack of a common definition and measure of adherence, which restricts the comparability of different studies, even though general themes and outcomes are consistent across the literature in this area. Self-reported adherence data may provide a deceptively positive view of actual daily practice, due to the fact that patients might have either forgotten treatment or do not want to admit nonadherence. This bias was minimized in this study by the fact that the participants were informed that their study results would not be shared with their ophthalmologists. Further information about the participants' intrinsic motivation and knowledge about the disease and treatment is needed to better understand the influence of basic health beliefs and health literacy nonadherence with prescribed therapies.

The proportion of participants reporting a switch in glaucoma medication before the survey was considerably lower than expected (36.3%) and generally observed in routine clinical practice. It is possible that many participants were not aware or did not recall that their treatment had been switched in the past. This highlights, once more, the need for greater patient engagement and ownership concerning management of self-administered medications. Future studies exploring the impact of treatment switch on nonadherence levels, particularly where medication is changed to a preservative-free formulation, would be of value and should include a review of the individual's clinical record or input from the treating physician to minimize reporting errors and confirm the accuracy of these data.

The prevalence and severity of side effects in the treatment of glaucoma need to be studied in more detail and, in particular, the impact of adverse events on the patients' behavior. Our findings reveal a potential adherence benefit for patients treated with preservative-free medication, which should be further investigated with more study subjects. Astonishingly, the proportion of patients on a mixed medication regimen that was only partially preservative-free was relatively high in our study. For future studies, it will be important to deepen the understanding of the physicians' perspective of nonadherence, side effects, and their perception of preservatives for the management of glaucoma.
